# Cross-Signal Contribution as a Challenge in LC-MS/MS Bioanalysis

**DOI:** 10.1021/acs.analchem.5c02508

**Published:** 2025-06-30

**Authors:** Anna Siemiątkowska, Katarzyna Kosicka-Noworzyń, Marta Karaźniewicz-Łada, Celine Park, Pavel Gershkovich, Leonid Kagan

**Affiliations:** 1 Department of Physical Pharmacy and Pharmacokinetics, 37807 Poznan University of Medical Sciences, Rokietnicka 3, 60-806 Poznań, Poland; 2 Department of Pharmaceutics & Center of Excellence for Pharmaceutical Translational Research and Education, Ernest Mario School of Pharmacy, 242612 Rutgers, The State University of New Jersey, 160 Frelinghuysen Road, Piscataway, New Jersey 08854, United States; 3 School of Pharmacy, University of Nottingham, University Park, Nottingham NG7 2RD, U.K..

## Abstract

Liquid chromatography−tandem mass spectrometry is one of the most sensitive and reliable techniques in the quantitative bioanalysis of small molecules, but it is not free of challenges and traps. This Tutorial focuses on the possible causes of cross-signal contribution, which is often neglected, with seven real-world case studies in which interferences of different origins were identified and related problems were solved. A flowchart is suggested that guides readers step-by-step to narrow down the plausible reasons for the unexpected peaks in their LC-MS/MS assays. The impact of cross-signal contribution on quantitative bioanalysis is also discussed, along with measures that can be undertaken when the phenomenon has been identified.

## Graphical abstract

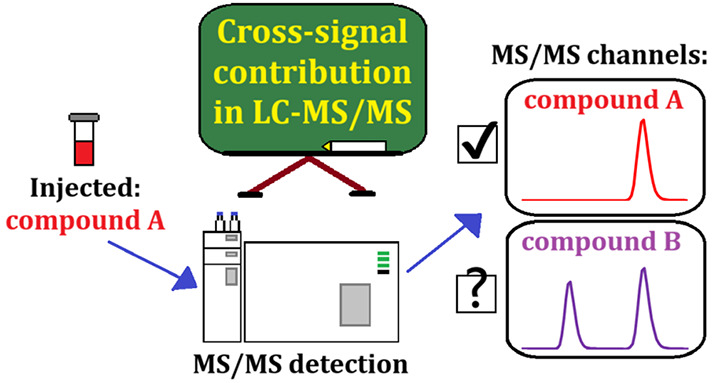

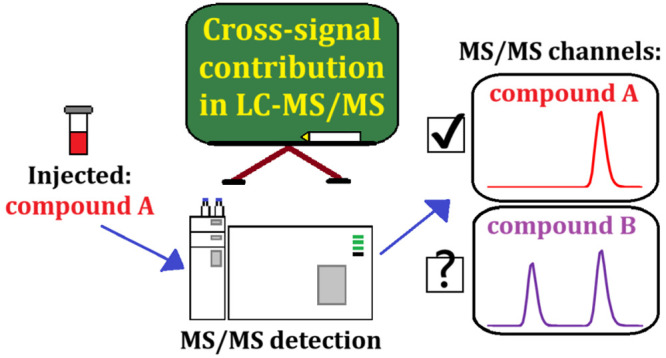

## Introduction

Liquid chromatography–tandem mass spectrometry (LC-MS/MS) is one of the most sensitive and selective techniques in the quantitative bioanalysis of small molecules,^1^ but it has its challenges. Multiple factors must be considered during method development,^2–4^ and a thorough validation is crucial for data quality.^5^ Cross-signal contribution is a lesser-known, challenging phenomenon, often neglected and unexplored. It was mostly reported between analytes and their stable isotope-labeled internal standards (SIL-ISs).^6–8^ However, more examples can be listed, so raising scientists’ awareness of this phenomenon is crucial to ensure good-quality data in published papers.

Notably, no clear definition of “cross-signal contribution” exists, as the term was not introduced in the validation guidelines by the major regulatory agencies (neither by the Food and Drug Administration, FDA,^9^ nor the International Council for Harmonization, ICH^10^). Although both agencies require confirming the purity of the SIL-IS, sufficient SIL-IS purity does not rule out cross-signal contribution. Furthermore, the guidelines address the method’s specificity, but a cross-signal contribution experiment between monitored compounds has not been suggested directly, which may leave some researchers unaware of the problem. In the literature, the “cross-signal contribution” term is usually directly interpreted as one analyte “contributing” to the other analyte’s signal and increasing the detector response.^6,7^ Thus, we propose that the “cross-signal contribution” in LC-MS/MS could be defined as *any unexpected peak in the compound’s MS/MS channels after an injection of another compound*.

In the past, a term frequently used in the context of cross-signal contribution was “cross-talk”, which referred to cross-signal contribution between analytes with similar product ions due to limitations of older mass spectrometers.^11^ However, unexpected peaks as a result of an in-source compound fragmentation were also classified as “cross-talk” by some researchers.^12,13^ This implies that the term was not limited to issues with emptying the collision cell and had a broader meaning in the LC-MS/MS community. Based on the definition provided by the Cambridge dictionary, “cross-talk” in electronics is interference in a communications system due to receiving the wrong signal. Thus, “cross-talk” in LC-MS/MS may refer to situations when the mass spectrometer misread the signals and recorded a response for the specific compound, even though this compound had not been injected.

Importantly, sometimes the interpretation of an incorrect signal may be difficult in LC-MS/MS. For example, imagine that the injected standard was contaminated—would detecting this contaminating compound really be incorrect in this case? In this context, the term “cross-signal contribution” seems to have a broader meaning, as the injected compound undoubtedly “contributed” to the signal in this contaminating compound’s MS/MS channel, even though the mass spectrometer read the signals correctly (albeit unexpectedly for us, as we did not suspect contamination). For this reason, in this Tutorial, we adopt the term “cross-signal contribution” rather than “cross-talk” to indicate all possible sources of the phenomenon. Of note, sometimes it may be impossible to decide if interferences are due to instrument limitations (so, a real “cross-talk”) or other reasons.

As cross-signal contribution is a relevant but often overlooked phenomenon, this paper highlights the importance of testing cross-signal contribution in LC-MS/MS. We discuss the possible causes of this undesired effect and share a series of real-world case studies in which we previously identified interferences of different origins. Finally, we suggest a flowchart that helps investigate a plausible source of cross-signal contribution in LC-MS/MS and assess its relevance for quantitative bioanalysis. Overall, this Tutorial provides a valuable framework for resolving cross-interferences in LC-MS/MS. To our knowledge, such a workflow has not been published before.

## Background

Let’s assume that the LC-MS/MS method enables the analysis of two compounds: A and B. We denote “A” for the compound being injected (as a single component) and “B” for the compound(s) supposedly absent in the injected solution and being monitored (e.g., imagine that we assess if an injection of the SIL-IS (A) produces a signal in the multiple reaction monitoring (MRM) channel of the analyte itself (B)).

The cross-signal contribution is defined as any unexpected peak in the MS/MS channel of B after injection of a supposedly pure solution of A. We do not discuss the “matrix effect” phenomenon, even though it results from one (or more) compound(s) affecting the other compound’s signal. However, in the context usually used in LC-MS/MS, the “matrix effect” causes signal suppression or enhancement, not additional peaks.^14^ When facing unexpected peaks, one should also investigate sample cross-contamination by the autosampler parts, which was ruled out in our examples. Therefore, in this Tutorial, we exclude “carry-over” and “matrix effect” as contributors to the observed phenomenon.

## Case Studies

The following bioanalytical challenges are illustrated and discussed in this Tutorial:

- **Case 1**: analysis of tryptophan (TRP) and its metabolites (kynurenine, KYN; kynurenic acid, KA; xanthurenic acid, XA) in the presence of SIL-TRP, TRP-D_5_ (Figure 1);^15^
- **Case 2**: analysis of metronidazole (MTZ) in the presence of SIL-MTZ, MTZ-D_3_ (Figure S1);
- **Case 3**: analysis of cefazolin (CFZ) in the presence of SIL-CFZ, ^13^C_2_,^15^N-CFZ (Figure S2);^16^
- **Case 4**: analysis of methadone (MTD) in the presence of SIL-MTD, MTD-D_3_ (Figure S3);^17^
- **Case 5**: analysis of azithromycin (AZM) in the presence of SIL-AZM, AZM-D_3_ (Figure S4);
- **Case 6**: analysis of rifampicin (RIF) and its derivative, rifampicin quinone (RIF-Q) (Figure 2);^18^
- **Case 7**: analysis of morphine (MOR) and its glucuronides (morphine-3-glucuronide, M3G, and morphine-6-glucuronide, M6G) in the presence of SIL-MOR, MOR-D_3_, and SIL-M3G, M3G-D_3_ (Figure 3).

**Figure 1:**
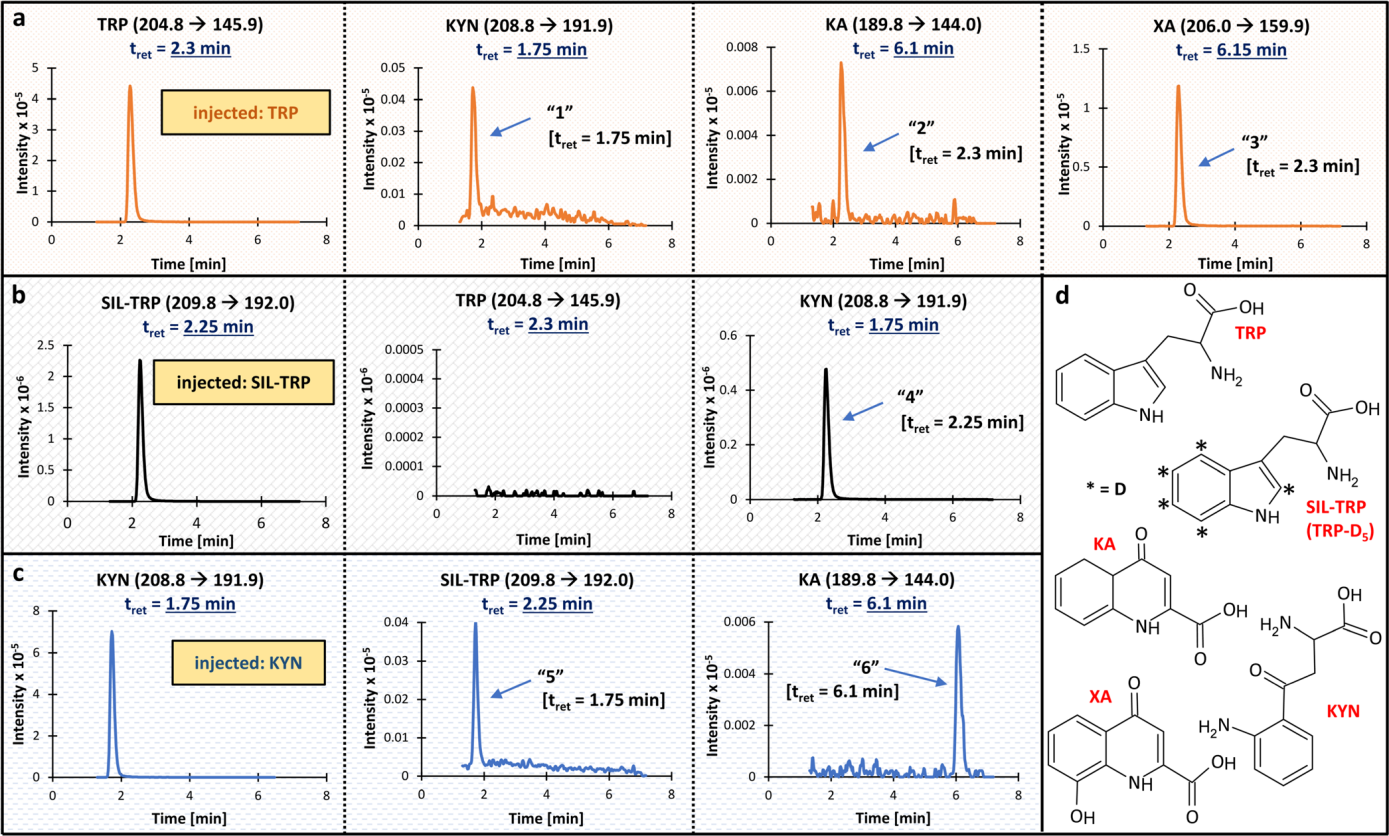
**Results of the cross-signal contribution experiment for Case 1.** Panels a–c present the MRM channels for **a**) TRP, KYN, KA, and XA after injection of pure TRP at ULOQ; **b**) SIL-TRP (TRP-D_5_), TRP, and KYN after injection of pure SIL-TRP (IS) at the concentration used in the study samples; and **c**) KYN, SIL-TRP, and KA after injection of pure KYN at ULOQ. Due to space constraints, only chromatograms with recorded interferences are presented, except for panel b (TRP pane), which confirms the purity of the SIL-IS. Panel **d** shows the structures of the analyzed molecules. The monitored MRM transition and expected retention time are provided for each compound. The unexpected peaks (interferences) are labeled 1–6, along with their retention times. No interferences were recorded in the TRP channel after injecting a high concentration of SIL-TRP (panel b). Abbreviations: IS, internal standard; KA, kynurenic acid; KYN, kynurenine; MRM, multiple reaction monitoring; SIL-TRP, stable isotope-labeled tryptophan; t_ret_, retention time; TRP, tryptophan; XA, xanthurenic acid; ULOQ, upper limit of quantitation.

**Figure 2:**
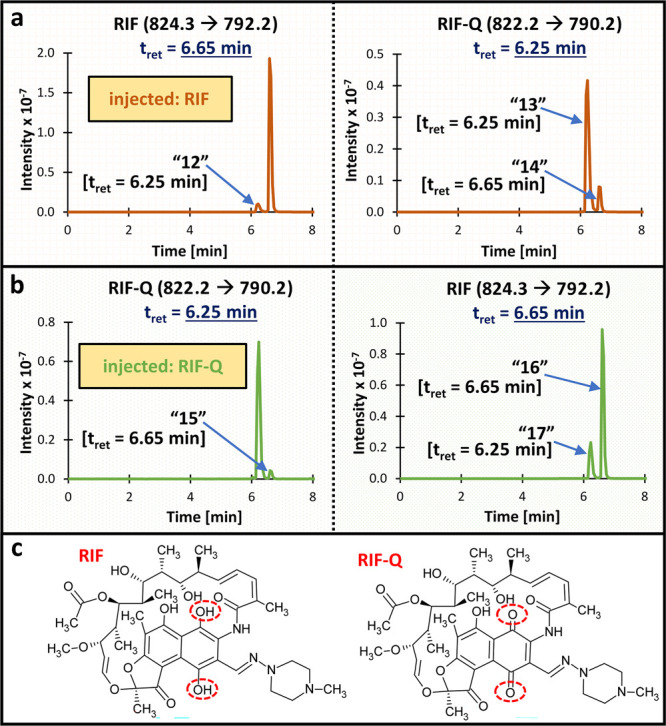
**Results of the cross-signal contribution experiment for Case 6.** Panels a–b present the MRM channels for **a)** RIF and RIF-Q after injecting a high concentration of RIF (no RIF-Q was added) and **b)** RIF-Q and RIF after injecting a high concentration of RIF-Q (no RIF was added). Panel **c** shows the structures of the analyzed compounds (structural differences are marked with red circles). The monitored MRM transition and expected retention time are provided for each compound. The unexpected peaks (interferences) are labeled 12–17, along with their retention times. Abbreviations: MRM, multiple reaction monitoring; RIF, rifampicin; RIF-Q, rifampicin quinone; t_ret_, retention time.

**Figure 3:**
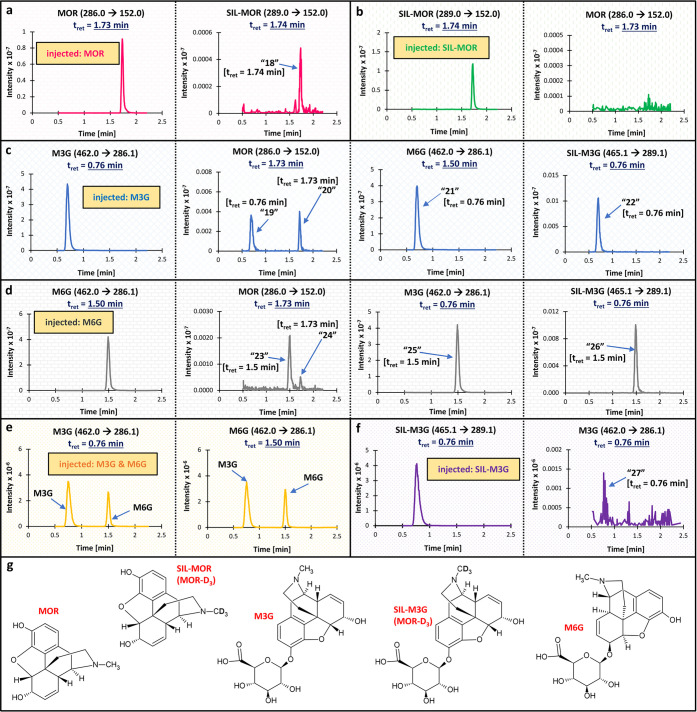
**Results of the cross-signal contribution experiment for Case 7.** Panels a–f present the MRM channels for **a)** MOR and SIL-MOR (MOR-D_3_) after injection of MOR at ULOQ (no SIL-MOR was added); **b)** SIL-MOR and MOR in a zero sample (no MOR was added); **c)** M3G, MOR, M6G, and SIL-M3G (M3G-D_3_) after injecting a high concentration of pure M3G (due to multiple interferences, the ULOQ for M3G was lowered in the final assay); **d)** M6G, MOR, M3G, and SIL-M3G after injecting a high concentration of pure M6G (due to multiple interferences, the ULOQ for M6G was lowered in the final assay); **e)** M3G and M6G at ULOQ (both M3G and M6G were present in the sample); and **f)** SIL-M3G and M3G in a zero sample (no M3G was added). Due to space constraints, only chromatograms with recorded interferences are presented, except for panel b (MOR pane), which confirms the purity of the SIL-IS. Panel **g** presents the structures of the analyzed molecules. SIL-MOR and SIL-M3G were used as the ISs in the assay. The monitored MRM transition and expected retention time are provided for each compound. The unexpected peaks (interferences) are labeled 18–27, along with their retention times. No interferences were recorded in the MRM channel of MOR after injecting SIL-MOR (panel b). Abbreviations: IS, internal standard; MOR, morphine; MRM, multiple reaction monitoring; M3G, morphine-3-glucuronide; M6G, morphine-6-glucuronide; SIL-MOR, stable isotope-labeled morphine; SIL-M3G, stable-isotope labeled M3G; t_ret_, retention time; ULOQ, upper limit of quantitation.

The examples come from the LC-MS/MS methods developed by our group over the past few years. Due to the nature of this Tutorial, we provide only general and relevant information on the LC-MS/MS methods. TRP,^15^ CFZ,^16^ MTD,^17^ and RIF methods^18^ have been described in detail elsewhere. Other methods have not been published.

Analyses were conducted on a QTRAP 6500+ (Sciex, Framingham, MA, USA) or an LCMS-8030 mass spectrometer (Shimadzu, Kyoto, Japan), operated in positive electrospray ionization mode with MRM acquisition. Tables S1 and S2 summarize information on the monitored Q1>Q3 transitions, retention times, and detected interferences. Information about the supplier and purity of the standards can be found in the Supporting Information.

## Flowchart

Based on the literature and our own experience, we distinguished three main situations when a cross-signal contribution can be encountered in LC-MS/MS:

- SIL-analyte contributes to the signal of the unlabeled analyte (SIL-analyte→analyte);
- unlabeled analyte contributes to the signal of the SIL-analyte (analyte→SIL-analyte);
- analyte contributes to the signal of another analyte (in a uni- or bidirectional way).

We found several aspects crucial for identifying the source and impact of the interfering peak, such as the analytes’ molecular weights, their structural similarities, compounds’ isotopic content, and the retention times of the analyte and the interference. Figure 4 presents a flowchart that facilitates the discovery of the potential causes of cross-interferences in LC-MS/MS. It includes examples from Cases 1–7 to illustrate better the characteristics of the cross-interfering compounds and their mutual relationship. Before using the diagram for their assays, readers are advised to complete a checklist (Table S4), which organizes the information about the analyzed compounds and observed interferences.

**Figure 4:**
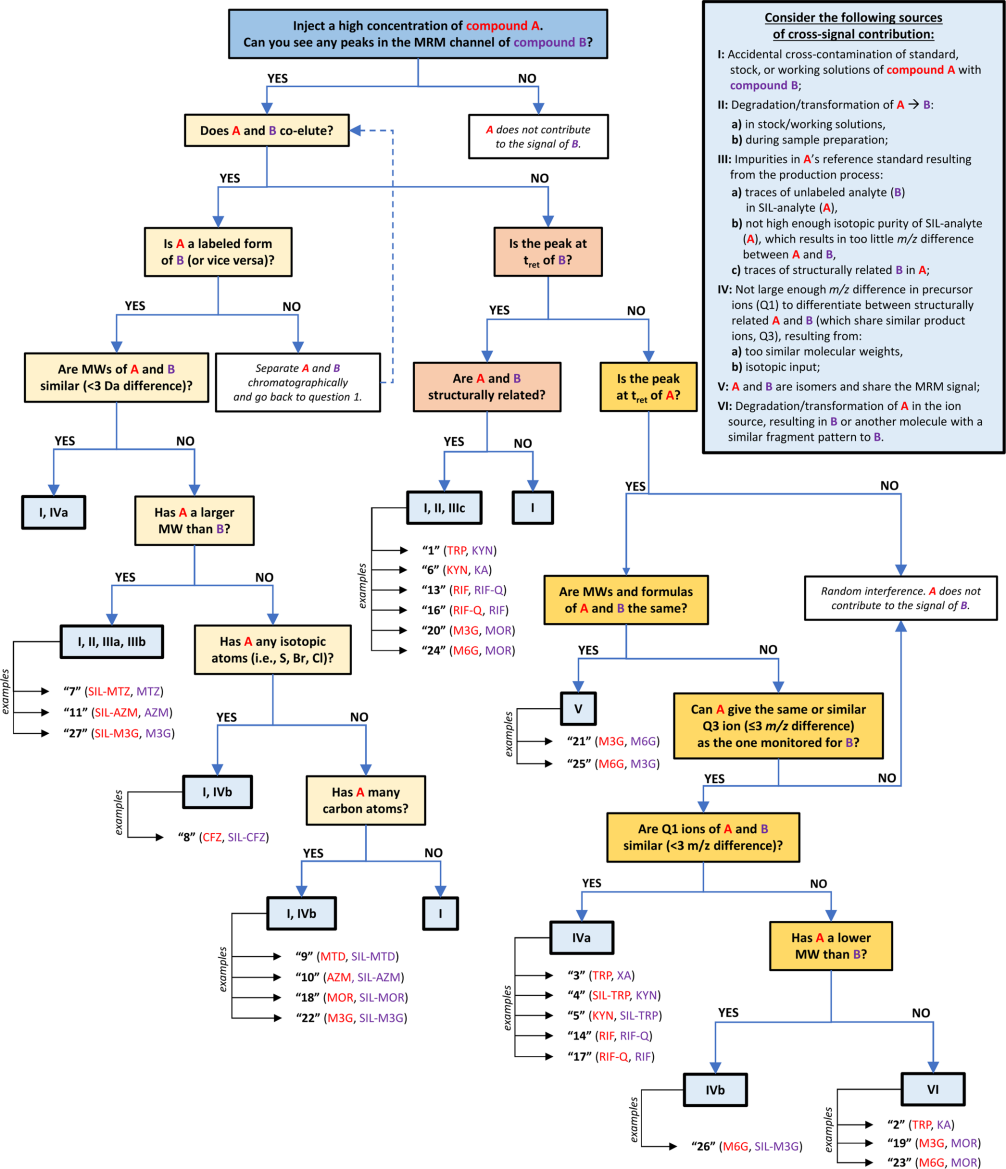
**Flowchart that helps to investigate the plausible causes of cross-signal contribution in LC-MS/MS.** Questions in yellow rectangles concern the analyte–SIL-analyte pair; questions in orange and red squares concern two chromatographically separated compounds for which the interference appears at the retention time of the injected (orange squares) or monitored compound (red squares). The answers (i.e., the plausible causes of cross-signal contribution) are presented in blue squares (numbers I–VI). Examples come from Cases 1–7 (peaks 1–27): injected compounds are marked in red, while monitored compounds are marked in purple. Abbreviations: A, injected compound; B, monitored compound; MRM, multiple reaction monitoring; MW, molecular weight; t_ret_, retention time.

## Sources of Interferences

### Contamination

If an injection of A generates a peak in the MRM channel of B at the retention time of B, then the interference most likely comes from compound B being present in the sample (unless A and B coelute; see answers I, II, and IIIc in Figure 4). It may indicate that stocks, working solutions, or standards of compound A are contaminated with B (accidentally, commercially, or resulting from the A>B transformation). If an injection of the SIL-analyte (commonly used as an SIL-IS in LC-MS/MS) produces the response for the unlabeled analyte, then insufficient purity of the SIL-analyte may be suspected (answer IIIa, Figure 4). Thus, the purity of the SIL-IS should always be assessed before the quantitative analysis.

In our assays, we sometimes observed insufficient purity of the SIL-IS, which required using another IS (peak “7”,Figure S1). However, in most methods, the unlabeled analyte was absent after injection of a high concentration of SIL-IS (e.g., Figures 1b, 3b, S2b, and S3b), or the detected level of interference was nonsignificant (≤20% of the analyte’s lower limit of quantitation, LLOQ) or could be easily corrected to a nonsignificant level by decreasing the SIL-IS concentration (peak “27”, Figure 3f). Sometimes, standard impurities cannot fully explain the presence of unlabeled species. For example, poor isotopic stability in water-containing solvents was proposed for ^13^C,D_3_-rofecoxib.^19^

Special attention is needed when simultaneously analyzing a parent compound and its metabolite(s) or other derivatives, as our experience with two drugs (MOR and RIF) and an endogenous compound (TRP) has shown. The standards of both MOR glucuronides, purchased as solutions, were contaminated with the parent drug (peaks “20” and “24”, Figure 3). To meet the validation guidelines,^9,10^ we lowered the upper limit of quantitation (ULOQ) for metabolites so that all interferences did not exceed 20% of the morphine’s LLOQ. Concerns about the M3G standard purity were previously reported.^20^ In the case of RIF (Figure 2), its standard (or solutions) clearly contained its derivative, RIF-Q (peak “13”); similarly, RIF-Q had detectable amounts of RIF (peak ”16”). Both RIF and RIF-Q were purchased as powders, and contamination was present even in fresh solutions, so it might have resulted from standard impurities or analytes’ interconversion in the solutions. As contamination was significant and would affect the method’s LLOQs, working solutions and calibration curves for RIF and RIF-Q were prepared separately.^18^ Standard impurity was also suspected as a root cause of peaks “1” and “6” in the TRP case (Figure 1). However, the level of interference for KYN and KA was acceptable (≤20% of the analytes’ peak areas at the LLOQs).^15^

### Isomeric Compounds

Tandem mass spectrometers identify analytes by their mass-to-charge ratio (*m*/*z*) on quadrupole 1 (Q1) and specific fragmentation patterns expressed by the *m*/*z* ratio on quadrupole 3 (Q3).^21^ Following this principle, if two compounds are structurally related and share the same molecular weights and formulas, then a cross-signal contribution between those analytes might occur.

Various examples can be listed when isomers were successfully analyzed by LC-MS/MS, including the determination of diastereoisomers (e.g., norpseudoephedrine–norephedrine or ephedrine–pseudoephedrine),^22^ *R*- and *S*-enantiomers (e.g., of salbutamol),^23^ or structural isomers (e.g., picolinic acid–nicotinic acid,^24,25^ bile acids^26^ or fentanyl analogs^27^). Each time, to obtain unbiased results, the compounds had to be separated on the column due to the shared MRM signals (answer V, Figure 4).

We have previously experienced the cross-signal contribution between isomers when analyzing tetrahydro and *allo*-tetrahydro metabolites of cortisol and cortisone^28^ or MOR glucuronides.^17^ Figure 3 illustrates the cross-signal contribution between the isomeric compounds, M3G and M6G. Both MOR glucuronides had the same precursor and product ions (462.0>286.1), so they could be identified only by their retention times.

### Similar Precursor and Product Ions

Even if the molecular weights of A and B are different, cross-signal contributions can still exist if compounds are structurally related and the difference in their masses is small (answer IVa, Figure 4). Tong et al.^29^ showed that substantial cross-signal contributions occurred when compounds had similar precursor ions (only 2 *m*/*z* difference) and the same (or very similar) product ions. Interferences were absent for different product ions (i.e., unrelated compounds), even if the precursor ions were similar. The minimal suggested difference in the molecular weights of the related coeluting compounds to avoid significant cross-signal contribution is 3 Da,^30^ although a larger difference (4–5 Da) was proposed by others.^3^ The criterion is rather arbitrary, which was demonstrated by Li et al.^31^ and Khamis et al.,^32^ who validated LC-MS/MS assays with only 2 Da differences between the analytes and their SIL-ISs and observed insignificant cross-interference. These two examples should be, however, treated as “exceptions that prove the rule”, as too-similar masses between related coeluting compounds usually cause problems in quantitative LC-MS/MS.

Particular attention should be paid to analytes for which metabolites with masses very close to that of the parent drug are suspected in biological samples; e.g., Furlong et al.^33^ deduced that the peak interfering with the SIL-IS (i.e., a labeled parent drug) came from the metabolite. We have observed a similar situation in the TRP assay (Figure 1), in which a D_5_-labeled TRP was used as the SIL-IS. One of the metabolites had a 4 Da larger molecular weight than the parent drug, resulting in a very close MRM transition to the SIL-IS (208.8>191.9 for KYN vs 209.8>192.0 for SIL-TRP). KYN generated an additional peak in the MS/MS channel of SIL-TRP (peak “5”), and SIL-TRP generated an additional peak in the channel of KYN (peak “4”). Notably, accurate measurements were feasible due to the chromatographic separation of the interfering compounds.

The TRP case also revealed one more cross-interference that resulted from similar MRMs: injecting TRP (204.8>145.9) caused an additional peak in the MRM channel of XA (206.0>159.9) at the retention time of the parent drug (peak “3”). The cross-talk could be explained by very similar precursor ions of TRP and XA (204.8 vs 206.0) and their similar fragment pattern. Even though the most intense product ions for TRP and XA seemed different (145.9 vs 159.9), the Q3 scan revealed that TRP gives an ion at *m*/*z* of 159, which was very close to XA’s ion at *m*/*z* of 159.9.^15^ This cross-interference could also be considered insignificant due to the different retention times of the compounds (Figure 1a).

We identified very similar precursor and product ions as one of the reasons for cross-signal contribution also in the case of RIF (824.3>792.2) and RIF-Q (822.2>790.2) (Figure 2). The compounds did not share the same MRM transitions, although the *m*/*z* differences were small. Injection of the RIF standard generated two peaks in the RIF channel—the expected one at the RIF’s retention time and an additional one (peak “12”) at the RIF-Q’s retention time. This observation had two sources: contamination of the RIF standard with RIF-Q (peak “12” showed up at the RIF-Q’s retention time) and the insufficient resolution of the mass spectrometer (detector could not distinguish very similar MRM transitions and recognized RIF-Q contamination as RIF despite the chromatographic resolution of the compounds). Additionally, the injection of the RIF standard generated two peaks in the RIF-Q channel—one peak at the RIF-Q’s retention time (peak “13”) and the other one at the RIF’s retention time (peak “14”). The peak “13” could be explained by standard impurities (RIF contaminated with RIF-Q). The peak “14” either was a result of very similar MRM transitions between RIF and RIF-Q and insufficient resolution of the mass spectrometer (the detector interpreted part of a RIF response as RIF-Q) or could be explained by the RIF>RIF-Q conversion in the ion source. Similar observations were made when the RIF-Q standard was injected (peaks “15–17”). However, in the case of RIF-Q, an isotopic input should also be considered (as described below), and no in-source fragmentation was assumed (discussed in a later paragraph) due to the lower mass of RIF-Q compared to RIF. In the case of RIF and RIF-Q, complete chromatographic separation of the analytes was the only way to ensure no impact of the interferences on the method’s accuracy.

## Isotopic Input

### Sulfur, Bromine, and Chlorine Atoms

Even if a mass difference between the analytes seems sufficient (but is relatively small), the cross-signal contribution may still be present due to the isotopic input (answer IVb, Figure 4). In fact, many elements, such as sulfur, chlorine, and bromine, are naturally abundant in isotopes having larger molecular weights than their most prevalent forms.^7^ For example, the isotopic ^34^S atom is naturally present in 4.3%, ^37^Cl 24.2%, and ^81^Br 49.3%.^34^ Thus, if analytes contain these elements, they are more prone to cross-interferences.^35^ This may be a source of inaccuracy in the case of the coeluting compounds, such as analytes and their SIL-ISs.

We have previously observed this type of interference when analyzing CFZ (with three sulfur atoms; Table S3). After injection of CFZ (454.9>323.0), a relatively large peak occurred in the MRM channel of SIL-CFZ (457.9>325.9) (peak “8”, Figure S2a), which forced us to use another IS.^16^ Other solutions have been proposed to mitigate the analyte→SIL-analyte cross-interference, such as monitoring the MRM transitions of the less abundant isotopes of the SIL-IS (which have larger *m*/*z* values),^7,35,36^ using a nonlinear calibration curve,^37^ or increasing the SIL-IS concentration.^36^ Notably, when the isotopic input is the only source of cross-interference between the analyte and its SIL-analyte, an additional peak is present only in the MRM channel of the SIL-analyte after injection of the unlabeled analyte, but not vice versa (compare Figures S2a and S2b). This is in contrast to the situations described above, where analytes had the same/similar MRM transitions.

### Carbon Atoms

Even the natural isotopic abundance of carbon may be a source of the cross-signal contribution in LC-MS/MS (despite only a 1.1% abundance of the ^13^C)^34^ when the analyte contains multiple carbon atoms.^38^ We have suspected this type of interference in Case 4 (peak “9”, Figure S3), Case 5 (peak “10”, Figure S4), and Case 7 (peaks “18” and “22”, Figure 3), in which the analytes contributed to their labeled counterparts. To our knowledge, no literature defines a cutoff for the number of carbon atoms that may cause such cross-interference. Whether such cross-signal contribution will appear and/or be significant depends on the method’s calibration range (specifically, on the unlabeled analyte’s ULOQ). It can be more of a problem in methods with a very wide calibration range. We recorded the analyte→SIL-analyte cross-signal contribution in analytes with about 20 carbon atoms (MOR, MTD, and M3G), though it was lower than for the compound with about 40 carbon atoms (AZM; Table S3).

The theoretical isotope distribution can be predicted using free online tools (e.g., https://www.sisweb.com/mstools/isotope.htm). In the above examples, the theoretical abundance of the analytes’ exact monoisotopic form was <85%, and their isotopic distribution showed a significant intensity of M+1 masses (Table S3). These isotopic species could contribute to the SIL-ISs’ signals and cause cross-interference. Importantly, in all of the presented pairs, the SIL-ISs had only 3 Da larger molecular weights than the unlabeled analytes. If the difference was larger, then the cross-signal contribution most likely would not have occurred.

The cross-signal contributions MTD→SIL-MTD and MOR→SIL-MOR were resolved by increasing the SIL-ISs concentrations, so the level of the interfering signal was reduced to a nonsignificant level (≤5% of the IS’s peak area).^9,10^ This approach was not feasible in the case of M3G→SIL-M3G due to contamination of the SIL-M3G standard with M3G (peak “27”, Figure 3). However, other interferences (i.e., contamination of the M3G standard with MOR; peak “20”, Figure 3) forced us to lower the ULOQ for M3G. This approach sufficiently reduced the M3G→SIL-M3G interference. In Case 5, another source of cross-interference was also detected (SIL-analyte→analyte, as described below), which ultimately hampered the use of AZM-D_3_ as an IS for AZM.

The isotopic cross-signal contribution may occur not only between the analytes and their SIL-ISs but also between structurally related analytes with close precursor and product ions. Accordingly, the isotopic input of ^13^C was suspected as one of the reasons for the shared MRM signals between RIF-Q and RIF (peak “17”, Figure 2). Apart from similar MRMs (discussed above), RIF-Q contains many carbon atoms (>40), resulting in a significant amount of naturally occurring M+1 species (Table S3). These isotopic species could lower the *m*/*z* difference between RIF and RIF-Q and cause cross-interference. An interesting (and unusual) situation is also presented in Figure 3 (peak “26”), where an analyte (M6G) generated a signal in the MRM channel of the labeled counterpart of its isomer (SIL-M3G). As interference appeared at the retention time of M6G and both compounds had similar precursor and product ions (only a 3 *m*/*z* difference), the isotopic input was suspected as a source of this interference. In examples of RIF and RIF-Q, and M6G and SIL-M3G, the chromatographic separation of the interfering compounds was the solution to the observed cross-signal contribution.

The importance of the naturally occurring ^13^C isotopes was revealed in the work of Staeheli et al.,^39^ who developed an LC-MS/MS method for multiple analytes (>80) in various tissues. The authors utilized ^13^C isotope monitoring for analytes present in the samples at high concentrations to avoid overloading the mass spectrometer. The procedure enabled the simultaneous analysis of compounds at very low and very high concentrations. At the same time, this showed that ^13^C isotopes generate a measurable signal in LC-MS/MS, so they cannot be ignored.

### Isotopic Purity of SIL-Analyte

In the case of SIL-analyte→analyte interference, the isotopic impurity must also be considered (answer IIIb, Figure 4), as our experience with the AZM assay showed (Figure S4). Unexpectedly, SIL-AZM significantly contributed to the signal of AZM (peak “11”), despite its high isotopic purity (≥99%) declared by the vendor. The cross-interference of SIL-AZM→AZM was so large that AZM-D_3_ could not be used as the SIL-IS for AZM (data unpublished). The certificate of analysis revealed that the reference standard of AZM-D_3_ contained <1% of the unlabeled analyte. However, the remaining content (≥99%) was broadly described as deuterated forms (D_1_–D_3_). Therefore, a too-high percentage of D_1_- and D_2_-forms (which led to too little mass difference between the analyte and its SIL-IS) was most likely the reason for the observed cross-interference.

## Degradation or Transformation in the Ion Source

Let’s consider a situation in which compounds A and B are well separated chromatographically. If the injection of A generates a signal in the MRM channel of B at the retention time of A, it may be due to A’s degradation (or transformation) to B in the ion source (answer VI, Figure 4). The interference at the retention time of A indicates that the interfering species (compound B) was absent from the sample during the injection. If B had been present, it would have been separated on the column, and the signal would have appeared at B’s retention time. These rule out contamination and indicate the interference developed in the mass spectrometer.

In-source fragmentation was previously described for various glucuronides,^40^ herbicides,^12^ or metanephrines.^13^ Moreover, other transformation processes, such as in-source cyclization^41^ and lactonization,^42^ were reported. Figure 3 shows the exemplary ion chromatograms of MOR glucuronides (M3G and M6G), which easily degrade in the ion source, generating the parent drug (MOR). In both examples, the injected metabolite caused two peaks in the MRM channel of MOR: “19” and “23” were due to the in-source fragmentation of the metabolite (as the interference appeared at the retention time of the metabolite), while “20” and “24” reflected contamination of the metabolite standard (or solution) with the parent drug (as the interference appeared at the retention time of the parent drug). The in-source transformation might also partly explain peak “14” that appeared in the RIF-Q channel at RIF’s retention time after injection of RIF (Figure 2). It could result from the RIF to RIF-Q transformation in the ion source. However, as explained above, other mechanisms should also be considered in the RIF→RIF-Q cross-signal contribution.

Degradation in the ion source was also suspected as a cause of cross-talk between TRP (204.8>145.9) and KA (189.8>144.0). After injection of a high concentration of TRP, an additional peak was detected in the MRM channel of KA (peak “2”, Figure 1). The Q1 mass spectrum confirmed that TRP degraded in the ion source and, except for the most intense ion at *m*/*z* of ∼205, resulted in peaks with smaller intensities, including the one at *m*/*z* of 188.^15^ As a result, TRP generated ions very similar to those of KA (only 2 *m*/*z* difference in both Q1 and Q3), and the mass spectrometer could not distinguish between TRP and KA.

The above examples were solved: either the interfering compounds were separated chromatographically (RIF and RIF-Q, TRP and KA, and MOR and M3G) or the method’s ULOQ and/or LLOQ were adjusted to make the interference insignificant. The second solution was used in the MOR assay, as MOR and M6G had similar retention times. In this method, we decreased the ULOQ for M6G (i.e., the degrading compound) and elevated the LLOQ for MOR (i.e., the compound created in the ion source). It resulted in only minor cross-interference in the MOR’s channel after injecting M6G’s ULOQ.

## Classical “Cross-Talk”

The cross-interference between analytes used to be attributed to instrument-related cross-talk, which might have occurred when compounds shared the same product ions and the collision cell was not cleared enough before the next compound was analyzed.^11^ The phenomenon could overestimate the results for the latter MRM transition if analytes were not separated chromatographically.^3^ The issue has been resolved in modern mass spectrometers; however, several measures could have been undertaken to mitigate the risk of the instrument-related cross-talk, including selecting different dominant product ions or increasing the interchannel delay period.^3^ Moreover, adding so-called “dummy transitions” (i.e., transitions that were meant not to monitor the specific compound but to separate the analytes with the same product ions)^11^ could be helpful in solving the problem. These supplementary transitions were used, e.g., by Mulvana et al.^43^ and Janda et al.^44^ in the early 2000s to separate the analytes from their SIL-ISs. The instrument-related cross-talk could also be overcome if the cross-interfering pairs were not monitored one after another (in this case, no additional transitions were added, but the order of monitored compounds was not random, and cross-interfering compounds had to be separated). This approach eliminated the classical cross-talk on the instruments on which it initially occurred, as demonstrated by Vogeser and Seger.^45^

## Impact on Quantitative Bioanalysis

Although the cross-signal contribution may occur for many reasons, not all interferences will impact the quantitative results to the same extent. Often, the problem can be solved by sufficient chromatographic separation. Then, the fact of cross-signal contribution will be irrelevant and will not compromise the method’s accuracy. However, the chromatographic separation is sometimes challenging, e.g., for the analyte–SIL-analyte pair, which (as a rule) coelute. Another situation occurs when working with the method for measurement of A and B: we inject pure A and see the interference, which we recognize as B. The compound B may be present in the injected solution due to standard A impurities or A→B degradation occurring before injection into the LC-MS/MS system (during sample storage or handling). Such interference can affect quantification.

In many cases, finding a standard of better purity or identifying the exact source of the interference will be impossible, so it must be ensured that the interference does not impact the results significantly. The acceptance criteria are specified by the FDA^9^ and EMA/ICH,^10^ which require all interferences detected at the retention time of the analyte to be ≤20% of the peak area at the LLOQ level and ≤5% for the IS. The final cross-signal contribution experiment should be performed in the matrix subsequently used in the assay, as some additional interferences may come from the matrix. Moreover, all monitored compounds should be injected at the highest utilized concentrations (so, the analytes at their ULOQs and ISs at the concentrations used during analysis), as multiple compounds may contribute to their peak areas, and a good method’s performance must be confirmed within the established calibration range.

Table 1 shows the suggested solutions for all cross-interferences mentioned in Figure 4.

**Table 1:** Suggested Solutions for All Sources of Cross-Signal Contribution (I–VI) Mentioned in Figure 4

No. of identified or suspected source of cross-signal contribution	Suggested approach
**I – Accidental cross-contamination**	• prepare new stock and working solutions
	• if suspect the standard’s contamination, obtain a new standard of A

**IIa – Degradation/transformation** **A→B** **in stock/working solutions**	• prepare fresh stock and working solutions
	• consider a different sample diluent for stock/working solutions
	• estimate carefully the stability of A and optimize the storage conditions

**IIb – Degradation/transformation** **A→B** **during sample preparation**	• consider using a different (less aggressive) sample preparation technique, e.g., lower temperature, different pH, light protection

**IIIa – Traces of unlabeled analyte in SIL-analyte, or** **IIIb – Insufficient isotopic purity of SIL-analyte**	• if possible, lower the SIL-analyte concentration so that the interference is nonsignificant^a^
	• if possible, increase the unlabeled analyte’s LLOQ so that the interference is nonsignificant^a^
	• if the level of interference is unacceptable, use a different SIL-analyte or use a structural analog as an IS

**IIIc – Traces of structurally related B** **in A**	• if possible, lower the ULOQ for A so that the interference is nonsignificant^a^
	• if possible, increase the LLOQ for B so that the interference is nonsignificant^a^
	• buy a new standard for A from a different vendor

**IVa – Too similar molecular weights of A and B, or** **IVb – Isotopic input**	If A and B are not the analyte–SIL-analyte pair:
	• A and B must be separated chromatographically unless cross-talk is nonsignificant^a^
	• if A and B coelute, consider decreasing the ULOQ for A and/or increasing the LLOQ for B so that the interference is nonsignificant^a^
	If A and B are the analyte–SIL-analyte pair:
	• consider using a different SIL-analyte (with more labeled atoms) or a structural analog as an IS unless the interference is nonsignificant^a^
	• if possible, increase the SIL-analyte concentration so that the interference is nonsignificant^a^
	• if possible, decrease the ULOQ for the analyte so that the interference is nonsignificant^a^
	• consider using for quantitation less abundant ions of SIL-analyte if they reduce or eliminate the cross-interferences

**V – A and B are isomers and share the same MRM signal**	• A and B must be separated chromatographically

**VI – Degradation/transformation of A in the ion source**	• A and B must be separated chromatographically unless the contribution is nonsignificant^a^
	• if A and B coelute, consider decreasing the ULOQ for A and/or increasing the LLOQ for B so the contribution of A to B is nonsignificant^a^
	• if A and B coelute, consider changing the mass spectrometer settings to lower the possibility of A transformation (e.g., decrease the ion source temperature, ion spray voltage, or collision energy for compound A)

a All interferences detected at the retention time of B must be ≤20% of B’s peak area at the LLOQ level if B is an analyte and ≤5% of B’s peak area in the samples if B is an IS.

## Cross-Interferences in Pharmacokinetic Studies

The possibility of metabolites generating a cross-interference in the parent compound MRM channel bears important implications in the assessment of the parent compound’s pharmacokinetics. This can be especially significant if chromatographic separation between the parent drug and the interfering metabolites is insufficient. Lack of knowledge about the existence of metabolites (especially during initial drug development), lack of therapeutic or toxic effects of known metabolites, or other factors (such as budgetary or time considerations) may lead to the development of a method that focuses solely on measuring the parent compound in biological samples. LC-MS/MS methods are considered very targeted and accurate due to monitoring the specific MRMs, and often chromatographic separation of compounds of interest is neglected. The resultant LC runs are often very short, and separation of the parent compound from metabolites may not be achieved (since such metabolites are not of interest and are not tested in the method). Furthermore, some metabolites may have much longer biological elimination half-lives and, therefore, accumulate to higher concentrations (e.g., in plasma) than a parent compound. Such a combination of conditions (cross-interference with the parent compound at the same/similar retention time and significant accumulation in the biological matrix over time) would result in the detection of falsely high concentrations of the parent compound, especially at the terminal phase of a pharmacokinetic profile. In turn, this would lead to the calculation of erroneous half-life and other pharmacokinetic parameters for the parent compound.

## Conclusions

Our work emphasizes the importance of considering the potential cross-signal contributions when developing an LC-MS/MS method. The sources of cross-interferences can vary greatly, and so can their impact on the quantitative bioanalysis. However, only problems noticed over time can be solved to ensure good data quality.

## Supplementary Material



## Notes

Data regarding LC-MS/MS methods presented in Cases 1–7: standards purity and source, monitored Q1 > Q3 transitions, retention times, chemical formulas, and isotopic distribution; chromatograms for Cases 2–5; a checklist to complete before working with a flowchart (PDF)
