# Supplementary material for: Cross-Signal Contribution as a Challenge in LC-MS/MS Bioanalysis
Source: Anal Chem. 2025 Jun 30;97(27):14077–87. doi: 10.1021/acs.analchem.5c02508 (PMC12268833; doi:10.1021/acs.analchem.5c02508)
Supplement: Supplementary file 1 [file ac5c02508_si_001.pdf]

Supporting Information

Cross-signal contribution as a challenge in LC-MS/MS bioanalysis

Anna Siemiątkowska [1,2], Katarzyna Kosicka-Noworzyń [1,2], Marta Karaźniewicz-Łada [1,2], Celine Park [2], Pavel Gershkovich[3], Leonid Kagan [2]*

[1] Department of Physical Pharmacy and Pharmacokinetics, Poznan University of Medical Sciences, 3 Rokietnicka Street, 60-806 Poznań, Poland;

[2] Department of Pharmaceutics & Center of Excellence for Pharmaceutical Translational Research and Education, Ernest Mario, School of Pharmacy, Rutgers, The State University of New Jersey, 160 Frelinghuysen Road, Piscataway, NJ 08854, USA;

[3] School of Pharmacy, University of Nottingham, University Park, Nottingham NG7 2RD, UK.

**corresponding author*

ANNA SIEMIĄTKOWSKA, MSc, PhD

e-mail: asiemiatkowska@ump.edu.pl

Table of Contents

Supplementary Data: Standards purity and source……………………………………………………………………………S3

Figure S1. Results of the cross-signal contribution experiment for Case 2…………………………………………………S4

Figure S2. Results of the cross-signal contribution experiment for Case 3…………………………………………………S4

Figure S3. Results of the cross-signal contribution experiment for Case 4…………………………………………………S5

Figure S4. Results of the cross-signal contribution experiment for Case 5…………………………………………………S5

Table S1. Retention times and monitored transitions of the compounds mentioned in Cases 1–7……………..S6

Table S2. Information on the interferences illustrated in Cases 1–7…………………………………………………………S7

Table S3. Formulas, masses, and isotopic distribution of analytes presented in Cases 1–7…………………………S8

Table S4. Checklist with the information regarding the injected compound A, the monitored compound B, and the detected interference helpful in finding the source of cross-signal contribution…………………………………S9

Supplementary Data: Standards purity and source

Case 1: L-tryptophan (Cat. No. T0254, purity ≥98%), L-kynurenine (Cat. No. K8625, purity ≥98%), kynurenic acid (Cat. No. K3375, ≥98%), and xanthurenic acid (Cat. No. D120804, purity 96%) were purchased from Merck/Sigma-Aldrich (Darmstadt, Germany). Tryptophan-D_5 (Cat. No. T947212, isotopic purity >95%) was purchased from Toronto Research Chemicals (North York, Kanada).

Case 2: Metronidazole (Cat. No. 9002409, purity ≥98%) was purchased from Cayman Chemical (Ann Arbor, MI, USA). Metronidazole-D_3 (Cat. No. M978800, isotopic purity >95%) was purchased from Toronto Research Chemicals (North York, Kanada).

Case 3: Cefazolin sodium (Alfa Aesar, Cat. No. J65274, purity 99.2%,) was purchased from Fisher Scientific (Fair Lawn, NJ, USA). $^{13}C_2,^{15}N$-cefazolin (Cat. No. 25319, purity ≥95%,) was purchased from Cayman Chemical (Ann Arbor, MI, USA).

Case 4: (±)-Methadone (Cerilliant, Cat. No. M-007, purity >99.9%) and (±)-methadone-D_3 (Cerilliant, Cat. No. M-021, isotopic purity [LOT# FE02042007]: D_0 – 0.01%, D_1 – 0.01%, D_2 – 1.27%, D_3 – 98.72%) were purchased from Sigma-Aldrich (St. Louis, MO, USA). Both standards were purchased as methanolic solutions and were stored according to the manufacturer's instructions.

Case 5: Azithromycin dihydrate (Cat. No. PZ0007, purity ≥98%) was purchased from Sigma-Aldrich (St. Louis, MO, USA). Azithromycin-D_3 (Cat. No. 20675, ≥99% deuterated forms D_1-D_3) was purchased from Cayman Chemical (Ann Arbor, MI, USA).

Case 6: Rifampicin (Cat. No. R3501, purity ≥95%) was purchased from Sigma-Aldrich (St. Louis, MO, USA). Rifampicin quinone (Cat. No. TRC-R508040, purity ≥95%) was purchased from Toronto Research Chemicals (North York, Kanada).

Case 7: Morphine (Cerilliant, Cat. No. M-005, purity >99%), morphine-D_3 (Cerilliant, Cat. No. M-006, isotopic purity [LOT# FE08281702]: D_0 – 0.0%, D_1 – 0.12%, D_2 – 5.89%, D_3 – 89.00%, D_4 – 4.99%), morphine-3β-D-glucuronide (Cerilliant, Cat. No. M-031, purity >99%), morphine-D_3-3β-D-glucuronide (Cerilliant, Cat. No. M-017, isotopic purity [LOT# FE07162006]: D_0 – 0.02%, D_1 – 0.11%, D_2 – 2.33%, D_3 – 97.54%), and morphine-6β-D-glucuronide (Cerillilant, Cat. No. M-017, purity 96.9%) were purchased from Sigma Aldrich (St. Louis, MO, USA). All standards were bought as ready-to-use solutions (different solvents, depending on the compound) and were stored according to the manufacturer's recommendations.

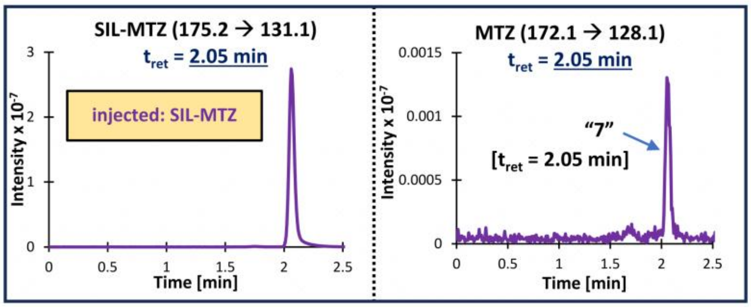

Figure S1. Results of the cross-signal contribution experiment for Case 2. Figure presents the MRM channels for SIL-MTZ (MTZ-D_3) and MTZ after injecting a high concentration of SIL-MTZ (no MTZ was added). SIL-MTZ was planned to be used as an IS in the assay (due to interference, it was not). The monitored MRM transition and expected retention time are provided for each compound. An unexpected peak (interference) is denoted with the number 7, along with its retention time. Abbreviations: MRM, multiple reaction monitoring; MTZ, metronidazole; SIL-MTZ, stable isotope-labeled metronidazole; t_{ret}, retention time.

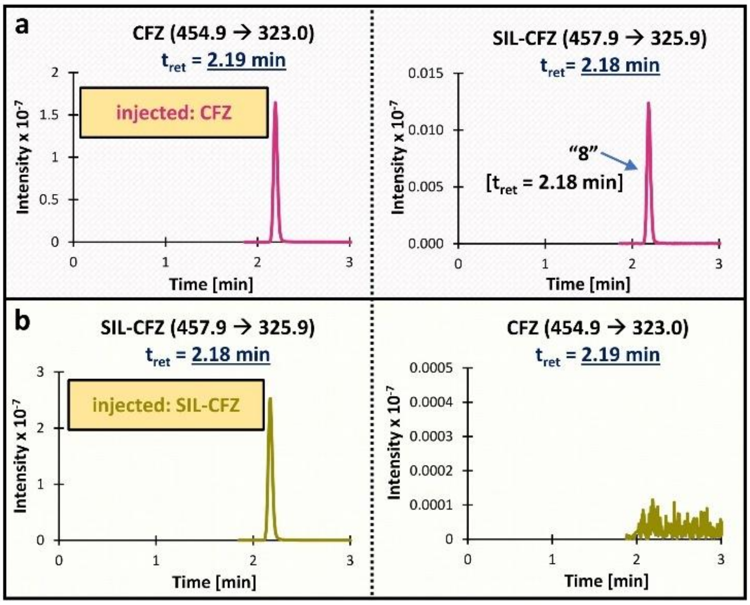

Figure S2. Results of the cross-signal contribution experiment for Case 3. Panels a-b present the MRM channels for **a)** CFZ and SIL-CFZ ($^{13}C_2$,^{15}N-CFZ) after injecting a high concentration of CFZ (no SIL-CFZ was added) and **b)** SIL-CFZ and CFZ after injecting a high concentration of SIL-CFZ (no CFZ was added). SIL-CFZ was planned to be used as an IS in the assay (due to interference, it was not). The monitored MRM transition and expected retention time are provided for each compound. An unexpected peak (interference) is denoted with the number 8, along with its retention time. No interferences were recorded in the CFZ channel after injecting a high concentration of SIL-CFZ (panel b). Abbreviations: CFZ, cefazolin; IS, internal standard; MRM, multiple reaction monitoring; SIL-CFZ, stable isotope-labeled cefazolin; t_{ret}, retention time.

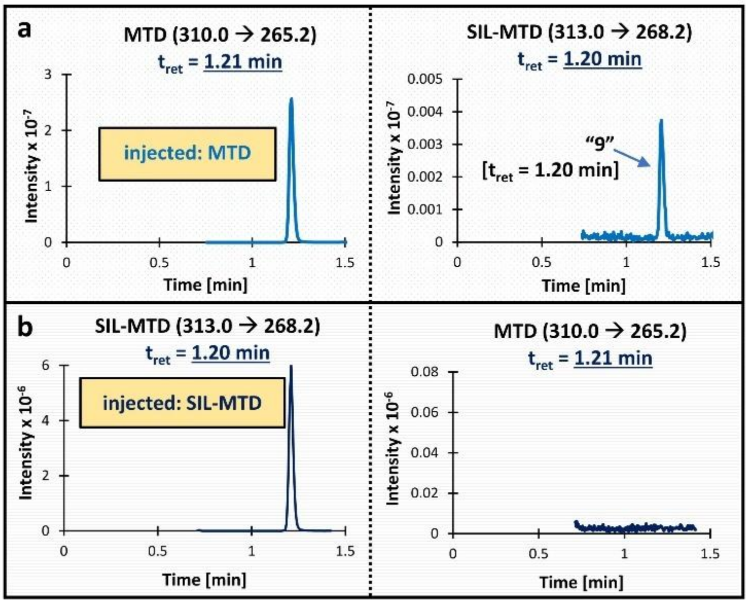

Figure S3. Results of the cross-signal contribution experiment for Case 4. Panels a-b present the MRM channels for **a)** MTD and SIL-MTD (MTD-D_3) after injection of MTD at the ULOQ level (no SIL-MTD was added) and **b)** SIL-MTD and MTD in a zero sample (no MTD was added). SIL-MTD was used as an IS in the assay. The monitored MRM transition and expected retention time are provided for each compound. An unexpected peak (interference) is denoted with the number 9, along with its retention time. No interferences were recorded in the MTD channel after injecting a high concentration of SIL-MTD (panel b). Abbreviations: IS, internal standard; MRM, multiple reaction monitoring; MTD, methadone; SIL-methadone, stable isotope-labeled methadone; t_{ret}, retention time; ULOQ, upper limit of quantitation.

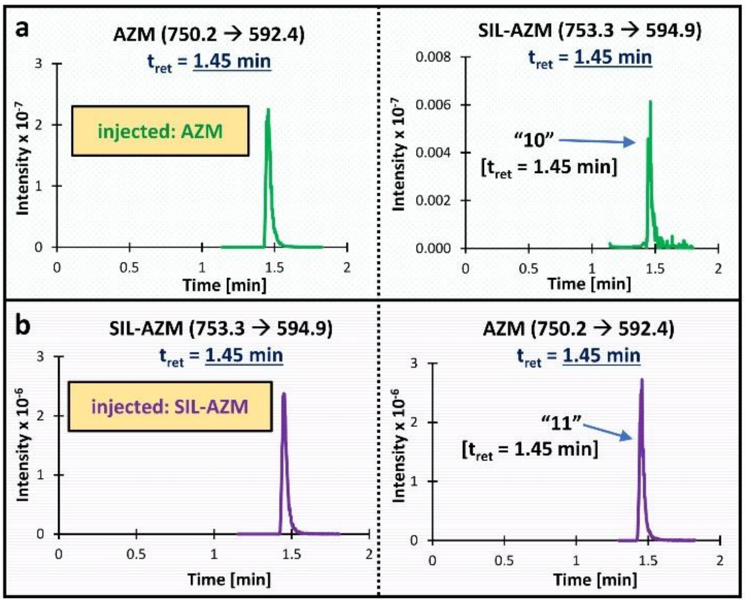

Figure S4. Results of the cross-signal contribution experiment for Case 5. Panels a-b present the MRM channels for **a)** AZM and SIL-AZM (AZM-D_3) after injecting a high concentration of AZM (no SIL-AZM was added) and **b)** SIL-AZM and AZM after injecting a high concentration of SIL-AZM (no AZM was added). SIL-AZM was planned to be used as an IS in the assay (due to interferences, it was not). The monitored MRM transition and expected retention time are provided for each compound. The unexpected peaks (interferences) are denoted with the numbers 10 and 11, along with their retention times. Abbreviations: AZM, azithromycin; IS, internal standard; MRM, multiple reaction monitoring; SIL-AZM, stable isotope-labeled azithromycin; t_{ret}, retention time.

Supplementary Tables

Table S1. Retention times and monitored transitions of the compounds mentioned in Cases 1–7

Case No.	Compound	Molecular weight [g/mol]	Precursor ion [*m/z*]	Product ions [*m/z*]	t_{ret} [min]
1	**TRP**	204.2	204.8	145.9; 118.0	2.30
	**SIL-TRP**	209.3	209.8	192.0; 150.0	2.25
	**KYN**	208.2	208.8	191.9; 94.0	1.75
	**KA**	189.2	189.8	144.0; 116.0	6.10
	**XA**	205.2	206.0	159.9; 132.0	6.15
2	**MTZ**	171.2	172.1	128.1; 111.4	2.05
	**SIL-MTZ**	174.2	175.2	131.1; 114.1	2.05
3	**CFZ**	454.5	454.9	323.0; 155.9	2.19
	**SIL-CFZ**	457.5	457.9	325.9; 156.1	2.18
4	**MTD**	309.4	310.0	265.2; 105.0	1.21
	**SIL-MTD**	312.5	313.0	268.2; 105.0	1.20
5	**AZM**	749.0	750.2	592.4; 158.0	1.45
	**SIL-AZM**	752.0	753.3	594.9; 158.0	1.45
6	**RIF**	822.9	824.3	792.2; 398.7	6.65
	**RIF-Q**	820.9	822.2	790.2; 397.6	6.25
7	**MOR**	285.3	286.0	152.0; 164.9	1.73
	**SIL-MOR**	288.4	289.0	152.0; 165.0	1.74
	**M3G**	461.5	462.0	286.1; 152.0	0.76
	**SIL-M3G**	464.5	465.1	289.1; 152.0	0.76
	**M6G**	461.5	462.0	286.1; 152.0	1.50

Abbreviations: AZM, azithromycin; CFZ, cefazolin; KA, kynurenic acid; KYN, kynurenine; MOR, morphine; MTD, methadone; MTZ, metronidazole; M3G, morphine-3-glucuronide; M6G, morphine-6-glucuronide; *m/z*, mass-to-charge ratio; RIF, rifampicin; RIF-Q, rifampicin quinone; TRP, tryptophan; SIL, stable isotope-labeled; SIL-AZM, azithromycin-D_3; SIL-CFZ, $^{13}C_2$,^{15}N-cefazolin; SIL-MOR, morphine-D_3; SIL-MTD, methadone-D_3; SIL-MTZ, metronidazole-D_3; SIL-M3G, morphine-D_3-3-glucuronide; SIL-TRP, tryptophan-D_5; t_{ret}, retention time; XA, xanthurenic acid.

Table S2. Information on the interferences illustrated in Cases 1–7

Case No.	Peak No.	Injected compound (A)	Monitored compound (B)	t_{ret} of interference [min]	Interference's t_{ret} matches t_{ret} of: A	B
1	1	TRP	KYN	1.75	-	✓
	2	TRP	KA	2.30	✓	-
	3	TRP	XA	2.30	✓	-
	4	SIL-TRP	KYN	2.25	✓	-
	5	KYN	SIL-TRP	1.75	✓	-
	6	KYN	KA	6.10	-	✓
2	7	SIL-MTZ	MTZ	2.05	✓	✓
3	8	CFZ	SIL-CFZ	2.18	✓	✓
4	9	MTD	SIL-MTD	1.20	✓	✓
5	10	AZM	SIL-AZM	1.45	✓	✓
	11	SIL-AZM	AZM	1.45	✓	✓
6	12	RIF [a]	RIF	6.25	- [a]	-
	13	RIF	RIF-Q	6.25	-	✓
	14	RIF	RIF-Q	6.65	✓	-
	15	RIF-Q [b]	RIF-Q	6.65	- [b]	-
	16	RIF-Q	RIF	6.65	-	✓
	17	RIF-Q	RIF	6.25	✓	-
7	18	MOR	SIL-MOR	1.74	✓	✓
	19	M3G	MOR	0.76	✓	-
	20	M3G	MOR	1.73	-	✓
	21	M3G	M6G	0.76	✓	-
	22	M3G	SIL-M3G	0.76	✓	✓
	23	M6G	MOR	1.50	✓	-
	24	M6G	MOR	1.73	-	✓
	25	M6G	M3G	1.50	✓	-
	26	M6G	SIL-M3G	1.50	✓	-
	27	SIL-M3G	M3G	0.76	✓	✓

[a] The interference origin was complex, but one of its reasons was contamination of RIF with RIF-Q; thus, RIF-Q could be treated as the injected compound (the interference's t_{ret} matched the t_{ret} of RIF-Q).

[b] The interference origin was complex, but one of its reasons was contamination of RIF-Q with RIF; thus, RIF could be treated as the injected compound (the interference's t_{ret} matched the t_{ret} of RIF).

Abbreviations: AZM, azithromycin; CFZ, cefazolin; KA, kynurenic acid; KYN, kynurenine; MOR, morphine; MTD, methadone; MTZ, metronidazole; M3G, morphine-3-glucuronide; M6G, morphine-6-glucuronide; *m/z*, mass-to-charge ratio; RIF, rifampicin; RIF-Q, rifampicin quinone; TRP, tryptophan; SIL, stable isotope-labeled; SIL-AZM, azithromycin-D_3; SIL-CFZ, $^{13}C_2$,^{15}N-cefazolin; SIL-MOR, morphine-D_3; SIL-MTD, methadone-D_3; SIL-MTZ, metronidazole-D_3; SIL-M3G, morphine-D_3-3-glucuronide; SIL-TRP, tryptophan-D_5; t_{ret}, retention time; XA, xanthurenic acid.

Table S3. Formulas, masses, and isotopic distribution of analytes presented in Cases 1–7

Case No.	Analyte	Chemical formula [a]	Exact mass [Da] (abundance, %) [b]	Isotopic distribution (relative mass intensity) [c,d]
**1**	**TRP**	$C_{11}H_{12}N_2O_2$	204.09<br>(87.4%)	204.09 (1.000)<br>205.09 (0.119)
	**KYN**	$C_{10}H_{12}N_2O_3$	208.08<br>(88.1%)	208.08 (1.000)<br>209.09 (0.108)
	**KA**	$C_{10}H_7NO_3$	189.04<br>(88.5%)	189.04 (1.000)<br>190.04 (0.108)
	**XA**	$C_{10}H_7NO_4$	205.04<br>(88.3%)	205.04 (1.000)<br>206.04 (0.108)
**2**	**MTZ**	$C_6H_9N_3O_3$	171.06<br>(91.8%)	171.06 (1.000)<br>172.07 (0.065)
**3**	**CFZ**	$C_{14}H_{14}N_8O_4S_3$	454.03<br>(70.6%)	454.03 (1.000)<br>455.03 (0.151)<br>456.02 (0.133)
**4**	**MTD**	$C_{21}H_{27}NO$	309.21<br>(78.6%)	309.21 (1.000)<br>310.21 (0.227)
**5**	**AZM**	$C_{38}H_{72}N_2O_{12}$	748.51<br>(62.9%)	748.51 (1.000)<br>749.51 (0.411)<br>750.51 (0.082)
**6**	**RIF**	$C_{43}H_{58}N_4O_{12}$	822.40<br>(59.2%)	822.40 (1.000)<br>823.41 (0.465)<br>824.41 (0.106)
	**RIF-Q**	$C_{43}H_{56}N_4O_{12}$	820.39<br>(59.2%)	820.39 (1.000)<br>821.39 (0.465)<br>822.40 (0.106)
**7**	**MOR**	$C_{17}H_{19}NO_3$	285.14<br>(81.8%)	285.14 (1.000)<br>286.14 (0.184)
	**M3G/ M6G**	$C_{23}H_{27}NO_9$	461.17<br>(75.4%)	461.17 (1.000)<br>462.17 (0.249)

[a] https://pubchem.ncbi.nlm.nih.gov;

[b] https://www.sisweb.com/referenc/tools/exactmass.htm;

[c] https://www.sisweb.com/mstools/isotope.htm;

[d] column presents isotopes with the highest mass intensities (relative occurrence was compared to the most prevalent isotope, for which mass intensity was equal to 1);

Abbreviations: AZM, azithromycin; CFZ, cefazolin; KA, kynurenic acid; KYN, kynurenine; MOR, morphine; MTD, methadone; MTZ, metronidazole; M3G, morphine-3-glucuronide; M6G, morphine-6-glucuronide; RIF, rifampicin; RIF-Q, rifampicin quinone; TRP, tryptophan; SIL, stable isotope-labeled; SIL-AZM, azithromycin-D_3; SIL-CFZ, $^{13}C_2$,^{15}N-cefazolin; SIL-MOR, morphine-D_3; SIL-MTD, methadone-D_3; SIL-MTZ, metronidazole-D_3; SIL-M3G, morphine-D_3-3-glucuronide; SIL-TRP, tryptophan-D_5; XA, xanthurenic acid.

Table S4. Checklist with the information regarding the injected compound A, the monitored compound B, and the detected interference helpful in finding the source of cross-signal contribution

☐	• **Molecular weight** [g/mol]	……………………………………………\ ………………………………………<br>compound A compound B
☐	• **Chemical formula**	……………………………………………\ ………………………………………<br>compound A compound B
☐	• **Q1 ion** [*m/z*]	……………………………………………\ ………………………………………<br>compound A compound B
☐	• **Q3 ions** [*m/z*]<br>(product ion scan)	……………………………………………\ ………………………………………<br>compound A compound B
☐	• **Retention time** [min]	………………………\ ……………………… \ ………………………<br>compound A compound B interference
